# Serum uric acid and diabetic peripheral neuropathy: a double-edged sword

**DOI:** 10.1007/s13760-022-01978-1

**Published:** 2022-05-29

**Authors:** Yong Zhuang, Huibin Huang, Xin Hu, Jinying Zhang, Qingyan Cai

**Affiliations:** 1 Department of Endocrinology, The Second Affiliated Hospital of Fujian Medical University, No.950 Donghai Street, Fengze District, Quanzhou City, Fujian Province China; 2 Department of Neurology, The Second Affiliated Hospital of Fujian Medical University, Quanzhou, 362000 China

**Keywords:** Type 2 diabetes mellitus, Diabetic peripheral neuropathy, Low serum uric acid

## Abstract

**Background and objectives:**

Research suggests that diabetic peripheral neuropathy (DPN) is related to high serum uric acid (SUA) level, although its correlation with low SUA level has not been reported. Here, diabetic patients with hyperuricemia were excluded, and the correlation between low SUA level and DPN was explored.

**Subjects and methods:**

This prospective observational clinical study enrolled 525 type 2 diabetes mellitus (T2DM) patients without hyperuricemia, who were divided into the diabetes with symptomatic neuropathy (150 cases), diabetes with asymptomatic neuropathy (125 cases) and diabetes with no neuropathy (250 cases) groups.

**Results:**

The SUA slightly decreased in subjects with asymptomatic DPN compared with those with no neuropathy and greatly decreased in subjects with symptomatic DPN compared with those without (*P* < 0.001). The association of the SUA with diabetic neuropathy was independent of the hyperglycemic state and other potential confounders (odds ratio 0.985 [0.981–0.988], *P* < 0.001). The SUA was closely correlated with the means of motor/sensory nerve amplitude and CV (all *P* < 0.001). The optimal cut-off point for SUA to distinguish patients with diabetic neuropathy from those without was 324 umol/L, with a sensitivity of 76.0% and a specificity of 79.2% (AUC = 0.806).

**Conclusions:**

The low SUA level is closely associated with DPN. Future studies are warranted to clarify the relationship.

**Supplementary Information:**

The online version contains supplementary material available at 10.1007/s13760-022-01978-1.

## Introduction

Diabetic peripheral neuropathy (DPN) is a common chronic complication of diabetes, with an incidence of 60% to 90%; nearly 50% of patients have no symptoms [1], and there is a high rate of disability. Early screening of risk factors can provide us with avenues for the development of new therapies for DPN [2].

So far, the factors related to the pathogenesis of DPN have not been understood completely. It is commonly accepted to be a multi-factor process and many hypotheses have been put forward, such as high condensation, duration of diabetes, hypertension, smoking, drinking, obesity, serum uric acid (SUA), et al. [3–6]. In recent years, research on the relationship between SUA and DPN is limited. Studies have found that DPN is closely related to elevated SUA level [3, 7, 8]. SUA is the product of purine catalysis by xanthine oxidase. High SUA could promote the migration of vascular smooth muscle cells and inhibit the release of NO by endothelial cells, resulting in vascular dysfunction and irreversible damage, resulting in tissue ischemia and impaired peripheral nerve function [9]. However, given the important antioxidant effect of SUA, maintaining a too-low SUA level in the long term might conversely expose diabetes mellitus patients to increased oxidative stress and neuropathy disorders [10]. Furthermore, to date, there has been no report on the relationship between low SUA and DPN. In addition, little data is available for Chinese individuals who face an increasing incidence of T2DM [11]. Therefore, we evaluated the relationship of the low SUA with diabetic neuropathy.

## Research design and methods

### General information

**Fig. 1:**
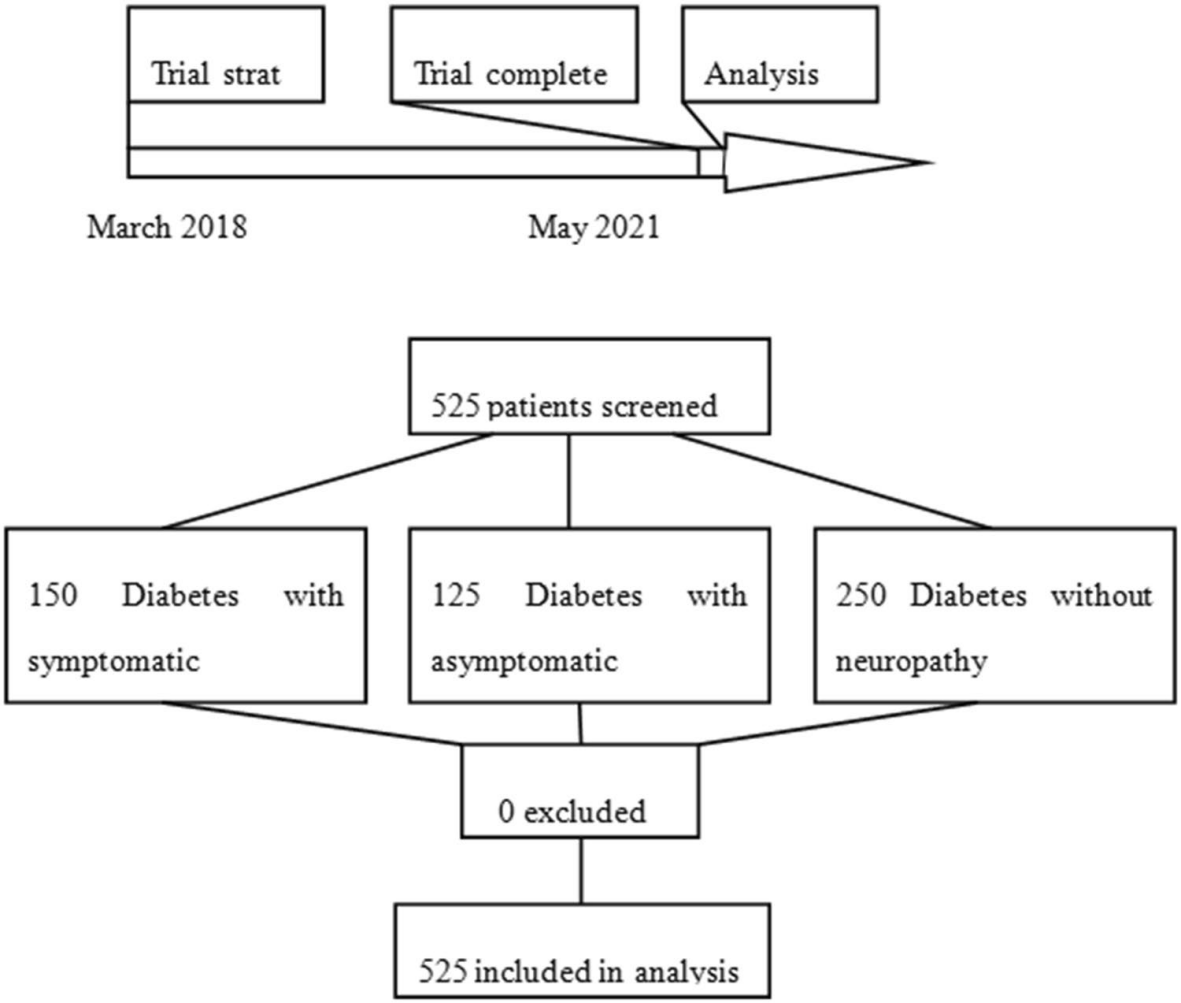
Flowcharts of the study. (In the trial, patient enrollment started in March 2018 and was completed in May 2021. Overall, 525 patients was further staged into three groups and analyzed.)

A total of 525 subjects who met the 1999 World Health Organization (WHO) type 2 diabetes diagnostic criteria and were registered consecutively as outpatients or inpatients with our hospital between March 2018 and May 2021 were randomly enrolled in the study (Fig. 1). All volunteers signed informed consent. The study was approved by the hospital and university scientific and ethics committees. The exclusion criteria were age < 20 years or > 75 years, hyperuricemia, gout, the use of any medication that might influence SUA (febuxostat, allopurinol, benzbromarone, diuretics, losartan) within one month, malnutrition, severe liver or kidney damage, trauma, surgery, tumor, acute infection, pregnancy or lactation, diabetic ketosis, blood disease, long-term alcohol abuse, other nondiabetic causes (such as cerebral infarction, neck lumbar disease, severe infection, poisoning, malnutrition, etc.) that could cause neurological damage, and other diseases that may be confused with the clinical symptoms of DPN, such as vitamin deficiency, osteoarthritis, peripheral vascular disease, and trauma surgery.

### Methods

#### Neurological symptoms and physical examination

Testing was performed on each participant by the same experienced physician according to standard procedure. All tests were conducted in a quiet laboratory. First, all patients had a complete history of neurological symptoms taken and were given a physical examination.

For somatic and cardioautonomic neuropathy, symptoms were documented, including numbness, paraesthesia, burning, deep aching, unsteadiness in walking, unexplained resting tachycardia and postural fainting [12].

The assessment by a professional medical staff member (Toronto clinical score) included 10 g nylon wire (pressure sense), tuning fork (vibration sense), temperature sense, acupuncture pain, and tendon reflex; positive findings on 2 of the 5 tests indicate abnormal signs of the nervous system [13, 14].

#### Nerve conduction velocity tests and clinical feature measurement

All patients were examined using an electromyography (EMG) instrument (Keypoint 9033A07, Denmark). All subjects were tested in a quiet environment. The motor conduction velocity (MCV) and motor nerve conduction amplitude (MNAP) of the median nerve, ulnar nerve, tibial nerve and common peroneal nerves were detected. The sensory conduction velocity (SCV) and sensory nerve action potential amplitude (SNAP) of the bilateral median nerve, ulnar nerve, superficial peroneal nerve and sural nerve were detected. The mean of MNAP was calculated using the formula: Amplitude motor nerve = [Amplitude (median nerve M) + Amplitude (ulnar nerve M) + Amplitude (tibial nerve M) + Amplitude (common peroneal nerve M)]/ 4. The mean of MCV, SNAP, and SCV were calculated respectively using the same method.

Body weight and upright height were measured on the same scales and wall-mounted stadiometer in light clothing without shoes before breakfast. Individual BMI was then calculated as weight (kg)/height (m)^2^. The right-arm blood pressure of each seated subject was obtained after 10 min of rest using a mercury sphygmomanometer. Retinal conditions were evaluated by ophthalmologists using a combination of clinical examination, stereoscopic retinal photographs, optical coherence tomography and fluorescein angiography.

All subjects stopped anticoagulant and antiplatelet drug use 2 weeks prior, and venous blood was collected in the morning from the antecubital vein after the subjects fasted for 10 to 12 h. SUA, fasting plasma glucose, serum creatinine, blood lipids, and liver and kidney function were measured by an automatic biochemical analyser (Cobas 8000; Roche, Germany). HbA1c was measured using high-performance liquid chromatography (D10; Bio–Rad, Berkeley, CA). Serum vitamin B12 (vit B12) was measured using an automated assay (Maglumi 4000; China). Platelet count (PLT) was measured by automated hematology analyzer (Sysmex XE-2100; China). The urinary albumin concentration was assessed using immunonephelometry (DCA2000; Bayer, Leverkusen, North Rhine-Westphalia, Germany). The urinary creatinine concentration was quantified using the alkaline picrate method. The individual urinary albumin-creatinine ratio (UACR) was then calculated as albumin (mg)/creatinine (g). Endogenous creatine clearance (Ccr) was calculated to estimate the glomerular filtration rate according to the Cockcroft equation: Ccr = {[140– age (years) × body weight (kg)]/[0.818 × serum creatinine (Scr, µmol/L)]} for males and × 0.85 for females.

#### Diagnosis and stages of polyneuropathy

Diabetic neuropathy was diagnosed according to the American Diabetes Association recommendation [15]. Polyneuropathy was further staged into three groups, diabetes with symptomatic neuropathy group, diabetes with asymptomatic neuropathy group and diabetes without neuropathy group.

### Statistical analysis

We used SPSS version 19 for statistical analysis. If some data sets are incomplete with respect to the values for some potential predictors, we may fill in the best estimates for the missing values, exploiting the correlation between variables in the data set (both predictor, endpoint, and auxiliary variables). Multiple imputation is a procedure to fill in missing values multiple times (typically at least five times) to appropriately address the randomness of the estimation procedure [16]. The data are expressed as the mean (SD) for normally distributed data. The chi-square test was used to compare the count data. Multiple comparisons among groups were assessed using one-way analysis and comparisons between two groups (LSD method) for variables. A t test was used for comparison between the two groups. SUA was later added to a logistic regression model, controlling for possible confounders. The relation of the SUA level to the nerve conduction (the mean of motor/sensory nerve amplitude/CV) was calculated using Spearman’s correlation analysis. Receiver operating characteristic (ROC) analysis was conducted with MedCalc Software version 19.0.4 to assess the accuracy of SUA level in distinguishing between patients with and without diabetic neuropathy. The optimal cut-off point was identified by calculating the area under the curve (AUC). *P* < 0.05 was considered indicative of statistical significance.

## Results

**Fig. 2:**
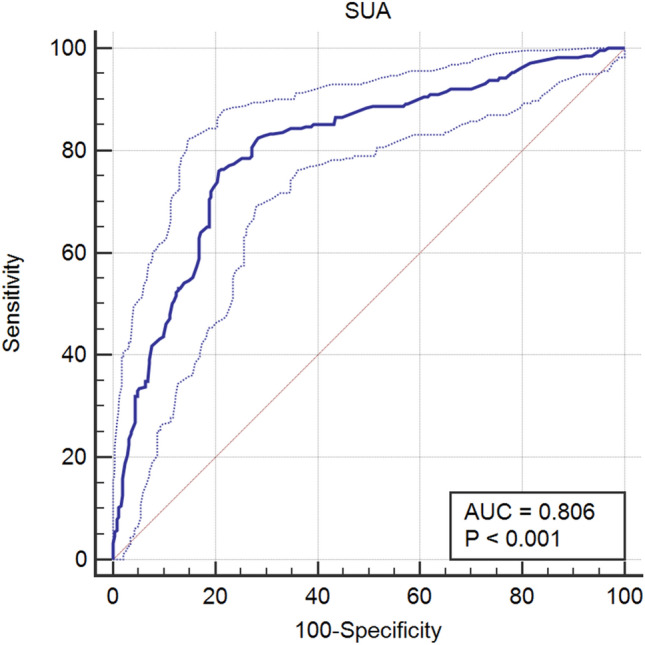
ROC curve of SUA

The study was completed by 525 subjects, including 250 diabetes without neuropathy, 125 diabetes with asymptomatic neuropathy and 150 diabetes with symptomatic neuropathy (Table 1). Among the three groups of subjects, there were no differences between any two groups in the following variables: age, sex ratio, BMI, blood pressure (SBP and DBP), blood lipids [total cholesterol, high-density lipoprotein (HDL) cholesterol and low-density lipoprotein (LDL) cholesterol], liver and kidney function [alanine transaminase (ALT), aspartate transaminase (AST), UACR and Ccr], vit B12 and PLT. The incidence of diabetic retinopathy was higher in the diabetes with symptomatic neuropathy group than in other groups. A comparison of diabetes among the groups revealed that that diabetes with symptomatic neuropathy group had the longest course of all groups (Table 1). SUA in all groups of diabetes was less than 420 (313.7 ± 73.1) umol/L. Moreover, the SUA was slightly reduced in the subclinical DPN group (*P* < 0.001) and further decreased in the confirmed DPN group (*P* < 0.001) (Table 2). Comparisons of nerve conduction studies parameters in the three groups, diabetes patients with symptomatic neuropathy showed the greatest impairment in the nerve conduction studies parameters including the mean of MNAP, MCV, SNAP and SCV (all *P* < 0.001) (Table 2). The SUA was further assessed in relation to neuropathy in a multivariate model, controlling for retinopathy and other covariables that may potentially influence the SUA level or neuropathy, including the disease course, age, HbA1c, estimated glomerular filtration rate (eGFR), UACR and vit B12. After adjustment, the SUA was still independently associated with diabetic neuropathy (odds ratio 0.985 [0.981 ~ 0.988], *P* < 0.001) (Table 3). Correspondingly, SUA level was positively correlated with the mean of MNAP, MCV, SNAP and SCV (*r* = 0.470, *P* < 0.001; *r* = 0.427 *P* < 0.001; *r* = 0.498, *P* < 0.001; *r* = 0.396, *P* < 0.001, respectively) (Table 4). The SUA level was shown to distinguish between patients with and without diabetic neuropathy. The optimal cut-off points were 324 umol/L for the SUA, with a sensitivity of 76.0% and a specificity of 79.2%, and the highest AUC equal to 0.806 (*P* < 0.001) (Fig. 2).

**Table 1:** Comparison of clinical features between different groups

Group	Diabetes without neuropathy	Diabetes with asymptomatic neuropathy	Diabetes with symptomatic neuropathy	*P* value
Case (male/female)	134/116	65/60	81/69	0.940
Age (years)	51.0 ± 8.6	51.2 ± 8.2	50.7 ± 7.8	0.889
Disease course (years)	6.6 ± 3.4	7.7 ± 2.9	8.7 ± 3.7	< 0.001
SBP (mmHg)	123 ± 9	121 ± 9	122 ± 9	0.141
DBP (mmHg)	69 ± 7	70 ± 6	70 ± 7	0.645
BMI (kg/m.^2^)	23.9 ± 1.7	24.0 ± 1.7	24.0 ± 1.7	0.854
FPG (mmol/L)	8.4 ± 1.4	8.2 ± 1.7	8.3 ± 1.7	0.422
HbA1c (%)	8.0 ± 1.5	8.3 ± 1.4	8.3 ± 1.3	0.085
TC (mmol/L)	4.8 ± 0.7	4.9 ± 0.6	4.8 ± 0.8	0.915
LDL-C (mmol/L)	2.8 ± 0.7	2.7 ± 0.9	2.7 ± 0.9	0.133
HDL-C (mmol/L)	1.4 ± 0.4	1.3 ± 0.4	1.4 ± 0.4	0.264
ALT (IU/L)	23 ± 3	23 ± 3	23 ± 3	0.239
AST (IU/L)	22 ± 3	21 ± 4	22 ± 4	0.168
Vit B12 (pmol/L)	540 ± 201	526 ± 203	525 ± 186	0.671
PLT (× 10.^9^/L)	238 ± 62	237 ± 64	243 ± 69	0.792
DR (%)	10.8	23.2	25.3	< 0.001
UACR (mg/g)	21.5 ± 3.2	21.9 ± 2.8	21.4 ± 3.1	0.368
eGFR [ml/(min·1.73 m.^2^)]	103.9 ± 26.9	105.1 ± 29.5	99.4 ± 26.7	0.169
SUA (umol/L)	353.2 ± 53.8	292.2 ± 70.6	265.8 ± 67.1	< 0.001

**Table 2:** Comparison of SUA, motor nerve and sensory nerve between groups

Group	Diabetes without neuropathy	*P* value^a^	Diabetes with asymptomatic neuropathy	*P* value^b^	Diabetes with symptomatic neuropathy	*P* value^c^
SUA	353.2 ± 53.8	< 0.001	292.2 ± 70.6	< 0.001	265.8 ± 67.1	< 0.001
*Motor nerve*						
Amp (mV)	9.42 ± 1.29	< 0.001	5.46 ± 1.72	< 0.001	4.22 ± 2.27	< 0.001
CV (m/s)	54.64 ± 2.14	< 0.001	43.69 ± 5.11	< 0.001	41.41 ± 5.35	< 0.001
*Sensory nerve*						
Amp (mV)	9.09 ± 1.38	< 0.001	4.64 ± 2.00	< 0.001	3.60 ± 2.20	< 0.001
CV (m/s)	54.29 ± 2.50	< 0.001	43.46 ± 6.64	< 0.001	40.35 ± 5.87	< 0.001

^a^*P*, diabetes without neuropathy vs. diabetes with asymptomatic neuropathy

^b^*P*, diabetes without neuropathy vs. diabetes with symptomatic neuropathy

^c^*P*, diabetes with asymptomatic neuropathy vs. diabetes with symptomatic neuropathy

**Table 3:** Multiple regression analysis of the relation of SUA to neuropathy

Covariables	*OR*	*95% CI*	*P* value
Disease course	1.089	1.012 ~ 1.171	0.022
Age	0.992	0.967 ~ 1.018	0.561
HbA1c	1.169	1.015 ~ 1.346	0.031
DR (%)	1.944	1.018 ~ 3.712	0.044
eGFR	0.998	0.991 ~ 1.006	0.624
UACR	1.001	0.937 ~ 1.071	0.966
Vit B12	1.000	0.999 ~ 1.001	0.830
SUA	0.982	0.978 ~ 0.985	< 0.001

**Table 4:** Correlation analysis between nerve conduction with SUA levels (Spearman correlation analysis)

Nerve conduction	*r*	*P* value
*Motor nerve*		
Amp (mV)	0.470	< 0.001
CV (m/s)	0.427	< 0.001
*Sensory nerve*		
Amp (mV)	0.498	< 0.001
CV (m/s)	0.396	< 0.001

## Discussion

DPN is one of the most common long-term complications of T2DM [17, 18]. Identifying and controlling the risk factors to prevent and slow the process of DPN is vital. However, when attention is paid to the harm brought by hyperuricemia to DPN, whether low SUA will also cause harm is often ignored.

The results of this study demonstrated that the low SUA is potential biomarkers of DPN. In this study, SUA was less than 420 umol/L in all subjects and the SUA in patients with DPN was significantly lower than those in diabetic patients without peripheral neuropathy. Furthermore, the SUA was changed in the early stage of diabetic peripheral neuropathy (diabetes with asymptomatic neuropathy). More importantly, the results showed both the amplitude and CV of motor/sensory nerves decreased with the decline of SUA level. It means that reduced SUA is positively associated with nerve damage.

DPN refers to clinical and/or electrophysiological evidence of peripheral neuropathy in patients with a confirmed diagnosis of diabetes, excluding other diseases. The incidence rate of DPN reported in China is as high as 85%. To date, although the pathogenesis of DPN remains unclear, previous studies suggest that DPN is associated with oxidative stress, microvascular disease, abnormal metabolic pathways, nerve growth factor, autoimmunity, and inflammation [19, 20]. Uric acid is the product of purine. According to the most recent international guidelines, consider hyperuricemia when SUA exceeds 420 umol/L. Previous studies have shown that hyperuricemia is closely related to DPN [7, 27]. Hyperuricemia could lead to vascular dysfunction, thrombosis and inhibition of NO release, thereby promoting the occurrence and development of DPN [9, 22]. However, uric acid, as a scavenger of reactive oxygen species and peroxynitrite, has important antioxidant effects [23, 24]. Ascorbate is recognized as a powerful antioxidant. In vitro, uric acid and ascorbate have similar antioxidant effect [25]. Therefore, long-term maintenance of too low uric acid concentration may conversely make diabetic patients more vulnerable to be exposed to oxidative stress, thereby increasing the incidence of diabetic neuropathy [10]. A meta-analysis from 4811 titles, 46 papers (*n* = 16,688 participants) showed that SUA was lower in dementia compared to controls [26]. In terms of animal experiment research, Huang TT [27] found that uric acid demonstrates neuroprotective properties for dopaminergic neurons in Parkinson's disease mice through modulation of neuroinflammation and oxidative stress. To some extent, as SUA has a strong hydrophilic antioxidant effect, potential neuroprotective properties may play an important role in neurodegenerative diseases [26]. Moreover, in this study, the SUA level were closely related to the degree of diabetic neuropathy; the SUA level were reduced in subclinical diabetic peripheral neuropathy and further decreased as the degree of diabetic peripheral neuropathy increased. This relationship was independent of covariables. In addition, when SUA was low, there were positive correlations between SUA and the amplitude and CV of sensory/motor nerves. Meaning that low SUA was strongly associated with nerve damage.

The relation between SUA level and neurologic disorders may be a double-edged sword. This has biological plausibility. Hyperuricemia has its harm, but low SUA could also have adverse effects. We should be aware of the lower limit of SUA. At the same time, this study concluded that the optimal cut-off point for the SUA level to distinguish patients with DPN from those without were 324 umol/L (≤ 324 umol/L). This cut-off point is within the reference range for SUA. Clinicians should be concerned about changes in SUA, which may be too low if the lower end of the SUA reference range is used as a standard.

## Conclusions

Based on the results in the present study, the low SUA level is closely associated with DPN. While clinicians are concerned about the risks of hyperuricemia, the appropriate level of uric acid control should also be considered. However, the sample size of this study was not large. Given the potential issues of confounding, further study of SUA and DPN is warranted.

## Supplementary Information

Below is the link to the electronic supplementary material.

Supplementary file1 (DOC 32 KB)

## Data Availability

The datasets used or analyzed during the current study are available from the corresponding author on reasonable request.
