# Supplementary material for: Serum uric acid and diabetic peripheral neuropathy: a double-edged sword
Source: Acta Neurol Belg. 2022 May 29;123(3):857–63. doi: 10.1007/s13760-022-01978-1 (PMC10238292; doi:10.1007/s13760-022-01978-1)
Supplement: Supplementary file 1 — Supplementary file1 (DOC 32 KB) [file 13760_2022_1978_MOESM1_ESM.doc]

1. Hosmer and lemeshow test of goodness-of-fit analysis

| *chi-square* | *df* | *P* value |
| --- | --- | --- |

| 8.455 | 8 | 0.390 |
| --- | --- | --- |

2. Multicollinearity test

| Covariables | *Tol* | *VIF* |
| --- | --- | --- |
| Disease course | 0.666 | 1.502 |
| Age | 0.974 | 1.027 |
| HbA1c | 0.990 | 1.010 |
| DR (%) | 0.687 | 1.457 |
| eGFR | 0.973 | 1.027 |
| UACR | 0.994 | 1.006 |
| Vit B12 | 0.990 | 1.010 |
| SUA | 0.961 | 1.041 |
